# Proceedings of the Esculapian Society of the Wabash Valley

**Published:** 1867-01

**Authors:** S. J. Young

**Affiliations:** Secretary


### PROCEEDINGS OF THE ESCULAPIAN SOCIETY OF THE WABASH VALLEY.

The Esculapian Society of the Wabash Valley met in annual session, at Kansas, Ill., October 31, 1866, at 2 P.M.

The Society was called to order by the President, Dr. JOHN TEN BROOK, of Paris.

The minutes of the last meeting being read and approved, the roll was called, and the following members, present, answered to their names:

Drs. John Ten Brook, Paris; James Steele, Grandview; W. M. Chambers, Charleston; O. Q. Herrick, John Mills, and Geo. Ringland, Kansas; Wm. Massie, Grandview; D. C. Jones, H. W. Davis, R. L. Walston, S. J. Young, S. B. Ten Brook, and A. J. Miller, Paris; G. W. Albin, Neoga; and J. Van. Dyke, Ashmore.

Dr. Massie, Chairman of the Committee on Indigenous Botany, read a very interesting report on that subject. On motion, the report was referred to the Committee on Publication.

On motion, the Society went into an election of officers for the ensuing year, which resulted as follows:—

*For President*—Dr. O. Q. HERRICK.
*For Vice-President*—Dr. WM. MASSIE.
*For Secretary*—Dr. S. J. YOUNG.
*For Treasurer*—Dr. R. L. WALSTON.

Dr. Chambers arose, and, addressing the Chair, delivered a brief eulogy upon the distinguished and lamented Prof. Brainard, and offered the following resolution:

*Resolved*, That a committee of three be appointed by the Chair, to draft suitable resolutions, expressive of the love and admiration we bear for the memory of this distinguished man, and report at the morning session.

The following gentlemen comprised the committee:—Drs. W. M. Chambers, H. W. Davis, and O. Q. Herrick.

The Society adjourned to meet at 9 o'clock A.M.

In the evening, the Society assembled at the Methodist Church, to listen to the annual address, delivered by Dr. R. L. Walston. The address gave evidence of careful research in its preparation, and was well received.

Nov. 1st, 1866.—The Society was called to order by the President, Dr. O. Q. HERRICK.

Minutes read and approved, and roll called.

Dr. R. L. Walston, by request, read a paper on typhoid fever, which was thoroughly discussed and referred to the Committee on Publication.

The committee (through their chairman) appointed to draft resolutions expressive of the sorrow of this Society for the death of Prof. Brainard, reported as follows:—

*Whereas*, The All-Wise Dispenser of Events has removed from his labors Prof. Daniel Brainard, thereby depriving our noble profession of one among its brightest ornaments, therefore,

*Resolved*, That, in his death, we recognize the hand of God, and bow in humble submission to his will; but, at the same time, we cannot but feel that a great light has gone out, because we esteem him as among the greatest intellects in our profession. His unsurpassed originality of thought enabled him to grasp the most intricate subjects; and his clear and convincing manner of explaining his views to his class, made him among the most useful of men.

*Resolved*, That, in consideration of the distinguished worth and ability of Prof. Brainard, the great teacher, we, his colaborers, desirous of manifesting our appreciation of his life and services, assist in the erection of a suitable monument to his memory.

On motion, the above report was referred to the Committee on Publication, with directions that they be requested to ask its insertion in the Chicago medical journals, *Cincinnati Lancet & Observer*, and the *Medical & Surgical Reporter*, Philadelphia.

The following preamble and resolution, offered by Dr. Chambers, were unanimously adopted:

*Whereas*, The Esculapian Society ranks as the oldest medical society in the State, extending over a large portion of it, and numbering among its members many prominent men of the profession who feel a lively interest in its prosperity, have concluded that, inasmuch as they contribute large numbers of students to the medical colleges of the State, it would not be presuming too much to make a recommendation for one of the Chairs made vacant by the death of the lamented Brainard. Therefore,

*Resolved*, That, in view of the fact, and the eminent qualification and fitness for such a position, we recommend the appointment of Henry W. Davis, M.D., of Paris, Illinois, to the Chair of Surgical Anatomy in Rush Medical College.

On motion, the fee bill adopted, and in use, by the Edgar Co. Medical Association, be, and is hereby adpoted by this Society.

The following gentlemen were appointed delegates to the State Medical Society:—Drs. James Steele, H. W. Davis, W. E. Morris, Geo. Ringland, Massie, Bridges, and Young.

To the National Association:—Drs. John Ten Brook, Massie, Herrick, Miller, Ringland, Young, Todd, Morris, Davis, S. B. Ten Brook, and Newell.

On motion, it was understood that the semi-annual meeting of this Society be held in Mattoon, beginning on the last Wednesday in May, 1867.

Standing committees were appointed.

Dr. H. W. Davis was appointed to deliver the public address.

After the usual votes of thanks for the use of the hall for our meeting, and especially for the generous reception we had met with at the hands of physicians and citizens, the Society adjourned.

S. J. YOUNG, *Secretary*.

